# Use of 3D prototyping in congenital cardiovascular diseases - initial experience in Hong Kong

**DOI:** 10.1186/1532-429X-17-S1-P225

**Published:** 2015-02-03

**Authors:** Janice J Ip, Peter K Hui, Robin Chen, Eddie Wu, KH Lau, Stephen Cheung, TC Yung, Wendy W Lam

**Affiliations:** Department of Radiology, Queen Mary Hospital, Hong Kong, China; Radiology Department, Baptist Hospital, Hong Kong, China; Department of Pediatrics-Cardiology, Queen Mary Hospital, Hong Kong, China; Department of Industrial and Systems Engineering, Hong Kong Polytechnic University, Hong Kong, China

## Background

The use of 3D prototyping in medical practice is becoming increasingly important, due to the development of 3D printing and the recent rapid advances in relevant techniques and equipments. The use of 3D model is especially important in the field of paediatric cardiology and congenital cardiovascular disease, where there is a clear advantage over conventional 2D images in demonstrating complex anatomies and relationships between different structures.

## Methods

The authors have selected a few paediatric cardiovascular diseases, and hope to share their initial experiences in 3D prototyping in this presentation.

## Results

Different cases are included to illustrate the use of 3D prototyping in management of congenital cardiovasclar diseases.

## Conclusions

The departments of radiology and paediatrics of a local teaching hospital and the department of medical engineering of a local university have started the first collaboration in Hong Kong, which pioneered the use of 3D printing in paediatric cardiovascular diseases.

**Figure 1:**
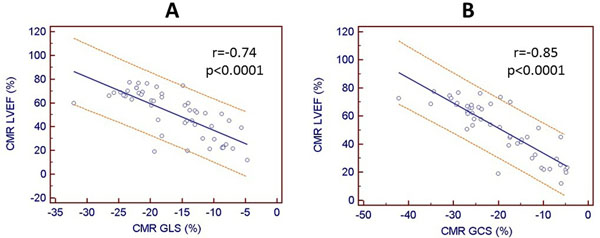
Figure 1

